# Molecular Characterization and Expression Patterns of the *HkSVP* Gene Reveal Distinct Roles in Inflorescence Structure and Floral Organ Development in *Hemerocallis fulva*

**DOI:** 10.3390/ijms222112010

**Published:** 2021-11-05

**Authors:** Yingzhu Liu, Yike Gao, Lin Yuan, Qixiang Zhang

**Affiliations:** 1 National Engineering Research Center for Floriculture, Beijing Key Laboratory of Ornamental Plants Germplasm Innovation & Molecular Breeding, Beijing Laboratory of Urban and Rural Ecological Environment, School of Landscape Architecture, Beijing Forestry University, Beijing 100083, China; liuyz@neau.edu.cn (Y.L.); Yuanlin0421@163.com (L.Y.); zqxbjfu@126.com (Q.Z.); 2 College of Horticulture and Landscape Architecture, Northeast Agricultural University, Harbin 150030, China

**Keywords:** floral, *SHORT VEGETATIVE PHASE*, MADS-box, *Hemerocallis*, yeast, overexpression

## Abstract

*SHORT VEGETATIVE PHASE* (*SVP*) genes are members of the well-known MADS-box gene family that play a key role in regulating vital developmental processes in plants. *Hemerocallis* are perennial herbs that exhibit continuous flowering development and have been extensively used in landscaping. However, there are few reports on the regulatory mechanism of flowering in *Hemerocallis*. To better understand the molecular basis of floral formation of *Hemerocallis*, we identified and characterized the *SVP*-like gene *HkSVP* from the *Hemerocallis* cultivar ‘Kanai Sensei’. Quantitative RT-PCR (qRT-PCR) indicated that *HkSVP* transcript was mainly expressed in the vegetative growth stage and had the highest expression in leaves, low expression in petals, pedicels and fruits, and no expression in pistils. The HkSVP encoded protein was localized in the nucleus of *Arabidopsis* protoplasts and the nucleus of onion epidermal cells. Yeast two hybrid assay revealed that HKSVP interacted with *Hemerocallis* AP1 and TFL1. Moreover, overexpression of *HkSVP* in *Arabidopsis* resulted in delayed flowering and abnormal phenotypes, including enriched trichomes, increased basal inflorescence branches and inhibition of inflorescence formation. These observations suggest that the *HkSVP* gene may play an important role in maintaining vegetative growth by participating in the construction of inflorescence structure and the development of flower organs.

## 1. Introduction

The transition from vegetative to reproductive growth is a major developmental switch in the life cycle of plants and is required for successful sexual reproduction of flowering plants. This process is controlled by environmental stimuli signals and the complex internal genetic network [1,2]. Molecular genetic analyses have identified three major genetic pathways *FLOWERING LOCUS T* (*FT*), *SUPPRESSOR OF OVEREXPRESSION OF CONSTANS 1* (*SOC1*), and *LEAFY* (*LFY*), that promote the floral transition in *Arabidopsis* [3,4]. The sequential interactions mediated by the above integrators constitute a conserved master regulatory pathway that controls vegetative phase changes in plants [5].

*SHORT VEGETATIVE PHASE* (*SVP*), which encodes a MADS-box transcription factor that represses the *FT* and *SOC1* floral pathways to suppress the transition of reproductive development, could be involved in maintenance of the plant vegetative growth period and determination of the specificity of the flower meristem [6,7,8,9,10]. In *Arabidopsis thaliana*, *SVP* is expressed broadly during vegetative development including in leaves and shoot apices [11]. It was reported that two paralogous genes, *SVP* and *AGAMOUS*-*LIKE 24* (*AGL24*), competitively bind *APETALA1* (*AP1*) to regulate the expression of *AGAMOUS* (*AG*), thereby affecting floral transition during the early stages of flower development [8,12,13,14]. Furthermore, *SVP* acts redundantly and directly to interact with *TERMINAL FLOWER1* (*TFL1*) in the establishment of floral meristems derived from the inflorescence meristem [15]. In addition, *SVP* mediates ambient temperature signalling by regulating *FT* expression [6] and responds to endogenous signals from the autonomous and gibberellin (GA) pathways to directly control *SOC1* transcription [16].

To date, several homologues of *SVP* have been characterized from different plant species including *Lycopersicon esculentum* [17], *Eucalyptus grandis* [10], *Medicago* L. [18], *Actinidia chinensis* [19], *Poncirus trifoliata* [9], *Hordeum vulgare* [20,21], *Brassica* L. [22], *Pharbitis nil* [23], *Paeonia suffruticosa* [24], *Narcissus tazetta* [25], *Antirrhinum majus* [26], *Prunus mume* [27] and *Mangifera indica* [28]. It has been reported that ectopic overexpression of *SVP* in transgenic *Arabidopsis svp* mutants leads to the formation of bud-like structures and leaf-like sepals instead of flowers [10,18], causes floral defects including delayed flowering in wild type (WT) *Arabidopsis* [18], and inhibits early transition and prolonged co-florescence development in tobacco [9]. These results suggest that plants have evolved diversified *SVPs* to address changes in species and environmental stress in terms of their roles in determining the vegetative phase and floral transition [12].

*Hemerocallis fulva*, commonly known as day lily, is an economically important landscape plant with a long history of cultivation in China [29]. The flowering and floral setting of *Hemerocallis* are valuable ornamental characteristics [30,31]. Little is known however about the floral transition of *Hemerocallis*, and the only flowering-related gene reported is *HkTFL1* [32]. In this study, we report the isolation and characterization of an *SVP*-like gene, named *HkSVP*, from *Hemerocallis fulva*. *HkSVP* was used for amino acid sequence phylogenetic analysis, expression profiling, protein integrates flowering signals, and functional analysis of overexpression in *Arabidopsis.* The data presented herein enrich our understanding of floral pathway integrators in *Hemerocallis*.

## 2. Results

### 2.1. Sequence Alignment and Phylogenetic Analysis

**Figure 1:**
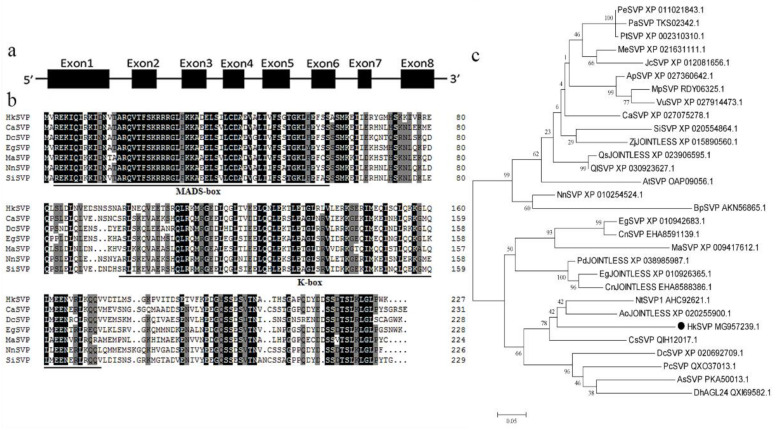
Schematic of the *HkSVP* gene structure and systemic phylogenetic relationship. (**a**) *HkSVP* CDS gene structure. Lines indicate introns, and black boxes indicate exons from the open reading frame. Numbers represent the exon or intron lengths in base pairs. The ATG translation initiation site and TAA translation stop site are marked. (**b**) Comparison of SVP amino acid sequences from selected species. Identical or conserved residues are indicated in black, similar residues are indicated in grey, and similar or weakly similar residues are indicated in white. The MADS-domain and K-domain are indicated by black lines. (**c**) Phylogenetic tree analysis of the amino acid sequence alignment of the *Hemerocallis* SVP protein with SVP proteins from other plant species. The trees were constructed by MEGA 7.0 software, using the minimum evolution phylogeny tested with 1000 bootstrap replicates, and the cut-off value was set to 50%. PeSVP (*Populus euphratica*, XP_011021843.1), PaSVP (*Populus alba*, TKS02342.1), PtSVP (*Populus* *trichocarpa*, XP_002310310.1), MeSVP (*Manihot esculenta*, XP_021631111.1), JcSVP (*Jatropha* *curcas*, XP_012081656.1), ApSVP (*Abrus precatorius*, XP_027360642.1), MpSVP (*Mucuna pruriens*, RDY06325.1), VuSVP (*Vigna unguiculata*, XP_027914473.1), CaSVP (*Coffea arabica*, XP_027075278.1), SiSVP (*Sesamum indicum*, XP_020554864.1), ZjJOINTLESS (*Ziziphus jujuba*, XP_015890560.1), QsJOINTLESS (*Quercus suber*, XP_023906595.1), QlSVP (*Quercus lobata*, XP_030923627.1), AtSVP (*Arabidopsis thaliana*, OAP09056.1), NnSVP (*Nelumbo nucifera*, XP_010254524.1), BpSVP (*Betula platyphylla*, AKN56865.1), EgSVP (*Elaeis guineensis*, XP_010942683.1), CnSVP (*Cocos nucifera*, EHA8591139.1), MaSVP (*Musa acuminata*, XP_009417 612.1), PdJOINTLESS (*Phoenix dactylifera*, XP_038985987.1), EgJOINTLESS (*Elaeis guineensis*, XP_010926365.1), CnJOINTLESS (*Cocos nucifera*, EHA8588386.1), NtSVP1 (*Narcissus tazetta*, AHC92621.1), AoJOINTLESS (*Asparagus officinalis*, XP_020255900.1), HkSVP (*Hemerocallis* ‘Kanai Sensei’, MG957239.1), CsSVP (*Crocus sativus*, QIH12017.1), DcSVP (*Dendrobium catenatum*, XP_020692709.1), PcSVP (*Paphiopedilum callosum*, QXO37013.1), AsSVP (*Apostasia shenzhenica*, PKA50013.1), DhAGL24 (*Dendrobium hybrid*, QXI69582.1).

The full-length *HkSVP* cDNA sequence was obtained from the total RNA of young *Hemerocallis* ‘Kanai Sensei’ leaves by RT-PCR. Oligo d(T)18 was used for mRNA reverse transcription, and the specific primers *HkSVP*-F/R for the conserved 5′ and 3′ UTRs were used separately for PCR amplification (GenBank accession No. MG957239.1). The open reading frame of *HkSVP* consisted of 684 bp and was predicted to encode a protein of 227 amino acids. The theoretical isoelectric point (pI) of *HkSVP* was 6.38, and the calculated molecular weight of *HkSVP* was 25.83 kDa. Analysis of the full-length cDNA and genomic DNA sequences of *HkSVP* indicated that there were eight exons in *HkSVP* (Figure 1a). The putative HkSVP protein contains a MIKC-II type MADS-box motif in the N-terminus and a K-box motif in the middle domains and shares high similarity with other SVP-related proteins (Figure 1b).

We performed phylogenetic tree analysis to better understand the genetic relationship between *HkSVP* and other reported *SVP*-like genes. As shown in Figure 1c, the evolutionary tree is divided into two different evolutionary groups. The *SVP* homologues of monocotyledons and dicotyledons are clustered into different branches, indicating that the *SVP* gene was highly conserved during evolution. HkSVP is clustered with *Narcissus tazetta*, *Asparagus officinalis*, *Crocus sativus* and *Dendrobium catenatum*, indicating that it is more closely related to monocotyledonous plants and shows high homology with genes from these plants. It is genetically distant from and shows low homology with genes from woody plants such as *Populus trichocarpa*, *Populus euphratica* and *Populus alba*. suggesting a conserved function with these evolutionarily closer *SVP* homologues.

### 2.2. Subcellular Localization of HkSVP

**Figure 2:**
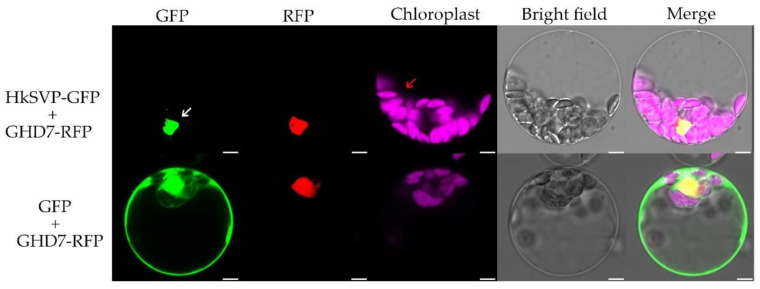
Subcellular localization of the HkSVP protein. PAN580-HkSVP-GFP and nuclear marker were transiently coexpressed in *Arabidopsis* protoplasts, and overlapping GFP and RFP signals were observed in the nucleus. White and red arrowheads indicate the nucleus and chloroplast, respectively. Scale bar, 5 µm.

To investigate the subcellular localization of HkSVP, we co-transformed the vector PAN580-HkSVP-GFP and nuclear marker gene pRFP-OsGHD7 [33] into *Arabidopsis* protoplasts derived from the WT. The colocalized fluorescent signals from GFP and RFP merged in the nuclear region, suggesting that HkSVP is a nucleus-localized protein (Figure 2). Furthermore, the pROKII-HkSVP-GFP fusion construct was transformed into onion epidermal cells using a gene gun. The green fluorescence signal of HkSVP-GFP was also distributed in the nuclear region (Figure S1). To confirm the confocal microscopy results and explore the subcellular location of HkSVP in more detail, we carried out analysis of the localization signals in the HkSVP amino acid sequence by using the WoLF PSORT tool (http://wolfpsort.org, accessed on 1 August 2019) [34]. The results showed that there was a predicted nuclear localization signal peptide in the N-terminal region with the PERIPHERAL motif, and that there was an XXRR-like membrane retention signal motif in the N-terminus along with a VREK motif, but other signal motifs for subcellular localization of the HkSVP protein were unexpectedly detected in the C-terminus region. The subcellular localization pattern indicated that the MADS-box protein HkSVP functions as a transcription factor in the nucleus.

### 2.3. HkSVP Interacts with the HkTFL1 and HkAP1 Proteins from Hemerocallis

**Figure 3:**
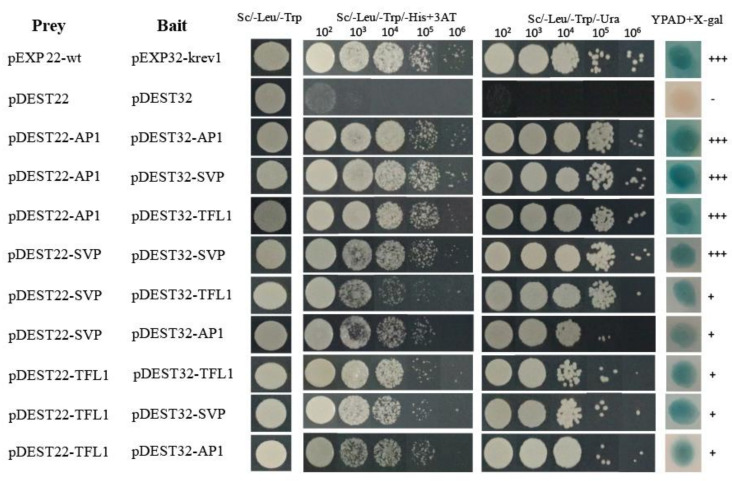
Interactions between HkSVP, HkTFL1 and HkAP1 proteins determined by a yeast two-hybrid assay. The positive and negative controls were pEXP22-wt/pEXP32-krev1 and pDEST22/ pDEST32 (Thermo, PQ10001), respectively. All hybrid yeast strains were spotted on selection plates. 10^2^–10^6^, fold dilution of the yeast liquid culture. +++, very strong interaction; +, strong interaction.

To further understand the functional interactions of HkSVP with other endogenous flowering-related proteins of *Hemerocallis* for floral development, the coding sequences of *HkSVP*, *HkAP1*, and *HkTFL1* from *Hemerocallis* were fused downstream of the GAL4 activation domain (AD) in the bait vector pDEST22, and other sequences were fused downstream of the GAL4 DNA-binding domain (BD) in the prey vector pDEST32. The BD and AD, cloned in the prey vector pDEST32 and bait vector pDEST22, respectively, were tested for their ability to interact with HkSVP. Analysis of the interactions of HkSVP with HkAP1 and HkTFL1 yielded positive results, with viable colonies on double drop out (Leu-Trp) medium (Figure 3). Through the self-activation experiment with the bait plasmid, it was concluded that 25 mM 3-aminotriazole (3AT) could be used as the screening concentration to inhibit the growth of false-positive yeast transformants (Figure S2). The interactions between these proteins and their interaction partners were further confirmed with the development of viable blue colonies on YPDA medium with the β-galactosidase induction assay (X-gal assay). The strength of interactions between the proteins was visually interpreted from the intensity of the colonies on triple drop out (Leu-Trp-His) and (Leu-Trp-Ura) medium, which was found to be the strongest for the interaction of AP1 with SVP and the weakest for the interaction of SVP with AP1. The interaction between the AP1 and SVP proteins has been reported in *Arabidopsis* [35], and AP1 was also shown to interact with TFL1 [36]. Therefore, we also performed a yeast two-hybrid assay using SVP and TFL1 from *Hemerocallis* to obtain insight into the functional similarity of the SVP proteins from *Hemerocallis* and *Arabidopsis*. The results of this analysis indicate that HkSVP can interact with both HkAP1 and HkTFL1. This suggested that the protein-protein interaction domains of MADS-box and PEBP proteins from *Hemerocallis* may interact specifically with related partners and might be conserved during floral induction and development among different species. Based on the data obtained in this study and that already available in the literature regarding protein-protein interactions among different species MADS-box genes [37], an interaction of the current state of the protein-protein interaction network is presented in Figure 3.

### 2.4. Expression Analysis of HkSVP in Hemerocallis

**Figure 4:**
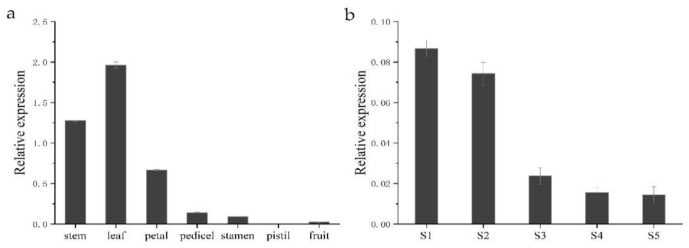
*HkSVP* expression analysis in *Hemerocallis* by qRT-PCR. (**a**) Stem, leaf, petal, pedicel, stamen, pistil and fruit. (**b**) S1–S5 stages of flower development. Error bars represent standard deviations calculated from three replications. The *Actin* gene was used as an internal control (Table S1).

To gain insight into the functional role of *HkSVP* in *Hemerocallis* development and the floral formation stage, we performed spatial analysis of the expression of the *HkSVP* gene using qRT-PCR in different organs and at different developmental stages. As shown in Figure 4, different expression patterns were found in these six organs. *HkSVP* mRNA transcript was principally found in stem and leaf tissues, with the highest expression in leaf tissues, but it was significantly expressed at low levels in the petal, pedicel and fruit and was almost undetectable in the pistil. Therefore, it appears that *HkSVP* was expressed during the early vegetative phase and increased towards the transition from the vegetative phase to reproductive phase. These observations were consistent with the results presented for *AtSVP* and showed that *HkSVP* played an important role in determining the duration of the vegetative phase [12].

To fully characterize the expression patterns of the *HkSVP* gene during the floral formation stage, we analysed the expression of *HkSVP* during the floral organ morphological structure stages from S1 to S5. The highest *HkSVP* expression was observed in the S1 petal differentiation stage, and then the expression began to decrease gradually until the lowest expression in the S5 stage, which was shown as S1 > S2 > S3 > S4 > S5. Based on these results, we conclude that a high level of *HkSVP* expression occurs in the early floral development stage, which might contribute to inducing floral formation and repressing flowering [12].

### 2.5. Ectopic Expression of HkSVP in Arabidopsis

**Figure 5:**
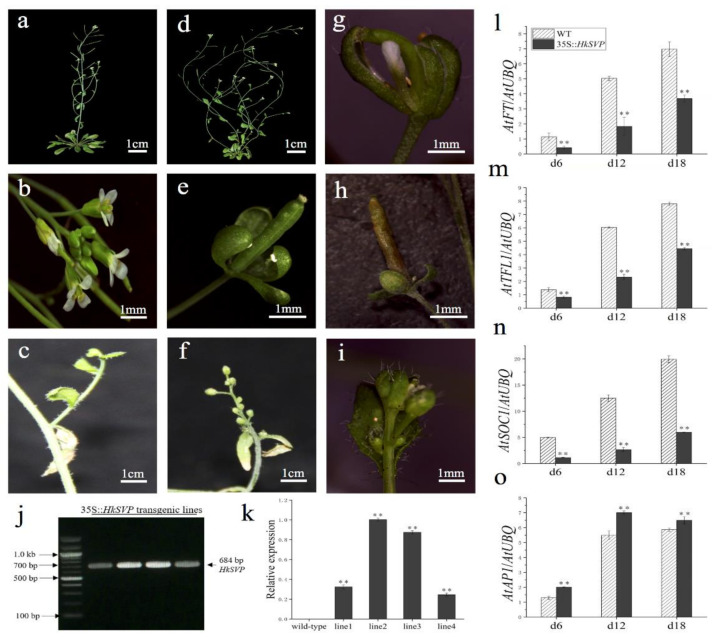
Phenotypes of p35S::*HkSVP* transgenic *Arabidopsis thaliana*. (**a**–**c**) Wild-type *Arabidopsis*. (**d**) Basal inflorescence branches increased. (**e**,**h**) Leafy sepals persistent. (**f**) The number of flower buds on the inflorescence of secondary branches increased. (**g**) Sepals abnormally enlarged. (**i**) Increased density of trichomes. (**j**) Results of an RT-PCR analysis of *HkSVP* expression in transgenic *Arabidopsis*. M, DL 2000 Marker; 1-4, independent 35S::*HkSVP* transgenic lines. (**k**) Results of qRT-PCR analysis of *HkSVP* transcript in transgenic *Arabidopsis* lines. Wild-type plants served as controls. (**l**–**o**) *AtFT* expression, *AtTFL1* expression, *AtSOC1* expression, and *AtAP1* expression. The error bars represent the standard deviation of the data obtained from three biological replicates. The expression levels of all the genes were normalized against *AtUBQ*-F and *AtUBQ*-R expression (Table S1). Values are presented as the mean ± standard deviation of three biological replicates. ** indicates significant differences (*p* < 0.01) according to Student’s test.

To assess the potential roles of *HkSVP* in the control of flowering time and regulation of flower development, the HkSVP protein was overexpressed in transgenic *Arabidopsis*. Four independent T1 generation transgenic lines were examined for kanamycin resistance, and the results were subsequently confirmed with RT-PCR and qRT-PCR analyses (Figure 5j,k). In the qRT-PCR data, line 1 and line 4 represent low level overexpression, line 2 and line 3 represent the highest level of overexpression of *HkSVP* in *Arabidopsis*. Lines 1, 2, 3, and 4 (L1, L2, L3, and L4) were screened using the selection method described above until T3 generation transgenic seeds were obtained. Four of the transformed *Arabidopsis* T3 lines had significantly delayed blooms under long-day growth conditions compared to WT plants during floral development (Table 1). At the onset of flower development, the transgenic plants were easily distinguished from WT Col, and flowers appeared at 35 days. However, the transgenic plants flowered after approximately 53 days (Table 1).

*HkSVP* affects the inflorescence structure of *Arabidopsis*. Compared with the WT (Figure 5a,b), transgenic lines 2 and 3 showed a significant increase in the number of typical basal branches (Figure 5d). At the same time, the plants also showed different degrees of abnormality in controlling the development of flower meristems. Leafy sepals are leathery in flower organs and reside at the base of developing pod (Figure 5e,h). The line 2 flower organ showed that the volume of sepals increased, and the abnormal flower structure with missing petals and stamens was wrapped in it (Figure 5g). Among the inflorescences formed on some secondary branches, WT *Arabidopsis* produced only a small number of flower buds (Figure 5c). In contrast, the number of flower buds on the inflorescence attached to the secondary branches of the transformed line increased significantly and grow alternately on the whole extended internode (Figure 5f). The density of trichome on flower buds and leaves of individual transformed lines was significantly higher than that of the WT (Figure 5i). In summary, *HkSVP* delayed flowering time in transgenic lines and as a floral repressor plays an important role in controlling inflorescence structure and flower organ morphology.

Furthermore, qRT-PCR was used to analyse the expression of flowering related genes (*AtFT*, *AtTFL1*, *AtSOC1*, *AtAP1*) in transgenic plants at different growth stages (6, 12, 18 days after seed germination) (Figure 5l–o). The results showed that the expression levels of *AtFT*, *AtTFL1*, and *AtSOC1* genes in transgenic plants were significantly lower than those in the WT, especially the *AtSOC1* gene was only weakly expressed in 35S::*HkSVP*. In contrast, the expression level of flower meristem determining gene *AtAP1* was consistent with that in the WT, and both lines showed a similar trend with the gradual increase in plant growth and development.

## 3. Discussion

We isolated the *HkSVP* gene from *Hemerocallis* ‘Kanai Sensei’ and examined its expression patterns and functions. Sequence analysis and multiple sequence alignment confirmed that *HkSVP* gene is similar to other *SVP* genes from different species. These genes contain MADS-box and K-box domains, which play a key role in DNA binding and protein complex formation [38]. Phylogenetic tree analysis showed that *HkSVP* is highly homologous to the *SVP* genes of monocotyledon plant species, and with the highest sequence similarity with *SVP* gene of *Narcissus tazetta*. The localization of HkSVP-GFP fusion protein in the nucleus also indicates that HkSVP may have transcription factor activity.

The interactions between the proteins revealed an important mode underlying the regulatory function. MADS-box transcription factors play a key role in plant flowering and its gene regulatory network [39]. As a flowering inhibitor, the *TFL1*-like gene in the PEBP family plays a key role in prolonging plant vegetative growth and delaying plant flowering [32]. Therefore, the study of the protein interaction between PEBP and MADS-box can lay a foundation for clarifying the molecular mechanism of the flowering regulation network. The results of this study show that the two-way hybridization between HkAP1, HkSVP, and HkTFL1 proteins can interact directly. It is speculated that they are jointly involved in various ways of regulating target genes, including the transformation from vegetative growth to reproductive growth, controlling the morphological structure of plants and the growth and development state of flower sequence.

Previous studies have indicated that the continuous flowering phenotype was related to the number of plant vegetative branches during hybridization of *Hemerocallis* in the field [29,32]. *HkSVP* expression has a broad expression pattern throughout various tissues of plants. The accumulation of *HkSVP* was higher in full leaves and stems, consistent with previous reports on *SVP* in *Arabidopsis* [12]. However, the expression levels of *HkSVP* decreased significantly during floral bud differentiation development. It is speculated that this gene plays an important regulatory role in controlling vegetative growth and transforming from vegetative growth to reproductive growth. A similar expression pattern was reported in an *SVP* homologue from another perennial plant, *Rubus idaeus* L. [40]. This pattern was consistent with the annual phase transition in woody plants, which does not occur in annual herbaceous plants.

*SVP* is a flowering repressor in *Arabidopsis* and reporting functionally delays flowering time. The overexpression of *SVP* genes from different species has been confirmed to cause alterations in flowering time and floral morphology [41,42,43]. In this research, *HkSVP* was found to improve the basal inflorescence branch of overexpressed transgenic *Arabidopsis*. Therefore, it was speculated that the *HkSVP* gene was primarily related to the control of the inflorescence branches of continuously flowering *Hemerocallis*. This result coincides with reports in transgenic *EgrSVP* and *PmSVP2* genes in *Arabidopsis* causing additional inflorescences [10,27], and the overexpression of *Narcissus tazetta NtSVP1* in *Arabidopsis* increased basal inflorescences [25]. However, 35S::*HkSVP Arabidopsis* showed abnormal floral development and a range of abnormal floral morphologies, such as abnormally enlarged sepals, persistent leafy sepals and an increased density of trichomes, which are similar to the phenotypes of *KiSVP*, *PtSVP*, and *EgSVP* overexpressing *Arabidopsis* plants [9,10,44]. At the same time, the ectopic expression of *HkSVP* enhanced the number of flower buds of every single inflorescence on the basal inflorescence. It is speculated that the *HkSVP* gene plays an important role in prolonging the single flower period and maintaining the vegetative growth of inflorescences. As the upstream regulator of the *TFL1* gene, *SVP* cooperates with the *SOC1*, *AGL24*, and *SEP4* genes to inhibit the expression of the *TFL1* gene to regulate the inflorescence branching pattern of *Arabidopsis* and further affect the expression of the *AP1* and *LFY* genes. This determines reproductive success, results from coordinated regulatory events mediated by both positive and negative regulators that consistently fine-tune the timing of flowering, and subsequent floral meristem development. Ultimately, this ensures that plants develop appropriate inflorescence architecture [15].

## 4. Materials and Methods

### 4.1. Plant Materials and Growth Conditions

The cultivar of *Hemerocallis* ‘Kanai Sensei’ was field grown at the Xiaotangshan Experimental Center of Beijing Forestry University (116.3°E,40.0°N, Beijing, China). The plant tissues, including stem, leaf, petal, pedicel, stamen, pistil and fruit were collected from the whole mature plant, as well as flower organs at different developmental stages and frozen in liquid nitrogen immediately and then stored at −80 °C before use. The five flower development stages S1–S5 were petal differentiation (S1), stamen differentiation (S2), pistil differentiation (S3), ovule and anther formation (S4), ovule and anthers maturity (S5) based on current report [32]. *Arabidopsis* ecotype Columbia (Col) and transgenic *Arabidopsis* plants were grown in a climate incubator (4000 lx) at 22 °C under long day conditions (16 h light/8 h dark).

### 4.2. Isolation of HkSVP

The cDNA sequence of *HkSVP* gene was identified from transcriptome of *Hemerocallis*. The full-length cDNA of the *HkSVP* was cloned from total RNA of *Hemerocallis* ‘Kanai Sensei’ young leaves by RT-PCR. The olig d(T)18 was reverse primer, and specific primers *HkSVP*-F and *HkSVP*-R (Table S1) were used to PCR amplification by FastPfu Fly DNA Polymerase (TransGen Biotech, Beijing, China), full-length sequences of the gene were obtained. The PCR products were purified and cloned into pEASY-Blunt Cloning Kit (TransGen Biotech) to do DNA sequencing.

### 4.3. Sequence Alignment and Phylogenetic Analysis of HkSVP

Alignment of cDNA and amino acid sequences were proceeded by BLAST (http://blast.ncbi.nlm.nih.gov/Blast, accessed on 11 June 2019). The conserved domains of *HkSVP* was predicted by Pfam version 29.0 software (http://pfam.xfam.org/, accessed on 11 June 2019). The full-length amino acid sequence of HkSVP protein in *Hemerocallis* was aligned with orthologs protein sequences from various plants through ClustalW2 software. The physical and chemical parameters of *HkSVP* were predicted by ProtParam (https://web.expasy.org/protparam/, accessed on 12 June 2019). The phylogenetic trees were constructed by MEGA 7.0 software, and bootstrap values were derived from 1000 replicates, and the cutoff value was set to 50%.

### 4.4. Quantitative Real-Time PCR (qRT-PCR) Analysis 

The tissue specific expression patterns of *HkSVP* from different developmental periods was analyzed by quantitative real-time PCR (qRT-PCR). For the spatial expression patterns of *HkSVP*, floral organs (petal, stamen and pistil), leaf, pedicel, stem and fruit were sampled. For the expression pattern of *HkSVP* in different flowering stages, floral bud were sampled at S1–S5 stage [32].

A one mg aliquot of total RNA treated with 5U DNase I (Invitrogen, Carlsbad, CA, USA) was used for reverse-transcription by PrimeScript RT Reagent Kit (TaKaRa, Beijing, China). Quantitative real-time RT-PCR (qRT-PCR) was carried out using CFX Connect System (Bio-Rad, Hercules, CA, USA) with SYBR Premix EX TaqTM II (TaKaRa). The specific primers *HkSVP*-QF and *HkSVP*-QR (Table S1) to detect the gene expression levels were designed by Primer Premier 5.0 software. *Hemerocallis Actin*-F and *Actin*-R gene were used to as an internal control [32]. To estimate the transcript levels of flowering-related genes in transgenic and wild-type *Arabidopsis* by qRT-PCR, aerial parts of different growth cycles *Arabidopsis* plants were harvested before inflorescences were formed. Specific primers for flowering-related genes were used for qRT-PCR, with the *AtUBQ* gene as an internal reference. All the primers used for qRT-PCR in the present study are listed in Table S1. Each sample had three biological replicates, while had three technical replicates for each biological replicate. The cycle threshold 2^−^^△△Ct^ based method was used to calculate the relative gene expression [45].

### 4.5. Subcellular Localization of the HkSVP

The pAN580-GFP and pROKII-GFP expression vector carrying the CaMV 35S promoter were digested with restriction enzymes XbaI and BamHI. The full-length *HkSVP* ORF (without a stop codon) amplified with primers *HkSVP*-SF and *HkSVP*-SR (Table S1) was ligated into the linearized pAN580-35S::HkSVP:GFP and pROKII-35S::HkSVP:GFP vector according to the homologous recombination method of the In-Fusion HD Cloning Kit (Takara). The construct without *HkSVP* (35S::GFP) was used as a control. The constructs were transformed into the *Arabidopsis* protoplasts according to established protocol [46]. The fusion of pAN580-HkSVP fluorescence and nuclear marker fluorescence was observed under laser confocal microscope (Zeiss LSM 710, Zeiss, Oberkoche, Germany). The tests of subcellular localization were repeated in onion epidermal cells. The pROKII-HkSVP-GFP fusion plasmid was transferred into onion epidermal cells by GJ-1000 Gene Gun System (Scientz, Beijing, China). The fluorescence signal was observed with a laser confocal microscope (Zeiss LSM 710).

### 4.6. Yeast Two-Hybrid Analyses

Protein interactions of HkSVP, HkAP1 (GenBank accession No. MG957240) and HkTFL1 (GenBank accession No. KY569646) were screened using the ProQuest two hybrid system (Invitrogen). Briefly, the coding sequence region of HkSVP, HkAP1 and HkTFL1 were constructed into pDEST22 as the prey vector, and the coding sequence region of HkSVP, HkAP1 and HkTFL1 were constructed into pDEST32 as the bait vector. pDEST33-SVP/AP1/TFL1 plasmid and pDEST22-SVP/AP1/TFL1 were co-transformed into MaV203 yeast cells. All protocols were carried out strictly according to the manufacturer’s user manual. Primers used are listed in Supplemental Table S1.

### 4.7. Generation of Transgenic Arabidopsis Plants

The coding sequencing reigon of *HkSVP* gene amplified with primers *HkSVP*-OE-F and *HkSVP*-OE-R was introduced into binary T-DNA vector pROKII by BamHI and KpnI, that was driven by the 35S promoter. The construct was transformed into Agrobacterium GV3101 competent cells using a freeze-thaw technique [47]. The *Arabidopsis thaliana* (Col-0) was used to transformation by the floral dipping method [48,49]. The resistant seedlings werer screened by 50 mg/L kanamycin-labeled and identified by PCR of *HkSVP* specific primers. The transgenic plants were transplanted into sterilized soil until harvest T3 generation seeds. Flowering time was measured by scoring the number of rosette leaves when the height of scape was 1 cm. All transgenic lines included 10 plants for statistics.

## 5. Conclusions

In this study, *HkSVP* gene was isolated and identified from *Hemerocallis*. The temporal and tissue expression analysis showed that the *HkSVP* was plays an important regulatory role in controlling vegetative growth and transforming from vegetative growth to reproductive growth. A yeast two hybrid assays showed that HkSVP protein could interact with the flowering-related proteins HkAP1, HkTFL1. Furthermore, *HkSVP* was found to improve the basal inflorescence branch of overexpressed transgenic *Arabidopsis*, and showed abnormal floral development and a range of abnormal floral morphologies. The data presented herein may provide new insights into the regulatory mechanisms of flower organ development and inflorescence structure of *Hemerocallis* and may be useful for comprehensive analyses of flower opening.

## Figures and Tables

**Table 1:** Flowering phenotypes of 35S::*HkSVP* in *Arabidopsis* under long-day conditions.

Genotype	Number of Rosette Leaves	Days to Flowering
Wild-type	12.4 ± 0.16	34.5 ± 1.12
Line 1	18.3 ± 0.44	44.1 ± 0.70
Line 2	24.2 ± 1.4 **	54.2 ± 1.31 **
Line 3	24.5 ± 1.0 **	52.5 ± 1.42 **

Values are presented as the mean ± standard deviation of three biological replicates. ** indicates significant differences (*p* < 0.01) according to Dunnett’s test. Error bars represent the standard deviation.

## Data Availability

No applicable.

## Supplementary Materials

The following are available online at https://www.mdpi.com/article/10.3390/ijms222112010/s1.
