# Supplementary material for: Molecular Characterization and Expression Patterns of the HkSVP Gene Reveal Distinct Roles in Inflorescence Structure and Floral Organ Development in Hemerocallis fulva
Source: Int J Mol Sci. 2021 Nov 5;22(21):12010. doi: 10.3390/ijms222112010 (PMC8585014; doi:10.3390/ijms222112010)
Supplement: Supplementary file 1 [file ijms-22-12010-s001.zip › ijms-1411063-supplementary.pdf]

International Journal of *Molecular Sciences*

MDPI

Supplementary Materials:

Table S1. Primers used in this study. Tables should be placed in the main text near to the first time they are cited.

Primer name	Sequence (5'→3')
HkSVP-F	ATGGTAAGGGAGAAGATACA
HkSVP-R	TCACTTCCATGGCAACCCTA
HkSVP-QF	TCTCTGGCACTGGCAAACTT
HkSVP-QR	GTCAAGAGATAGTTGTTCTC
HkSVP-SF	TCTAGAATGGTAAGGGAGAAGATACA
HkSVP-SR	GGATCCCTTCCATGGCAACCCTAAC
HkSVP-OE-F	GGATCCATGGTAAGGGAGAAGATACA
HkSVP-OE-R	GGTACCTCACTTCCATGGCAACCCTA
Actin-F	CTCAGTGGTGGGTCTACTAT
Actin-R	GAGGAGCAACCACCTTAATC
AP1-YF	ACAACTTTGTACAAAAAAGTTGGATGGGGAGAG-GAAAAGTG
AP1-YR	ACAACTTTGTACAAGAAAGTTGGGTTACTTAC-CATTCAAATG
TFL1-YF	ACAACTTTGTACAAAAAAGTTGGATGGCAAGAG-CAGTACAGCC
TFL1-YR	ACAACTTTGTACAAGAAAGTT-GGGTTAGCGCCTTCGCGCTGCAG
SVP-YF	ACAACTTTGTACAAAAAAGTTGGATGG-TAAGGGAGAAGATACAG
SVP-YR	ACAACTTTGTACAAGAAAGTT-GGGTTACTTCCATGGCAACCCTAAC
AtFT-F	GGTTGTTCCAGTTGTAGCAGG
AtFT-R	GGTGGAGAAGACCTCAGGAACT
AtTFL1-F	TGGATCGTTACAAACATTCCCG
AtTFL1-R	GTCTTTGCTTCTGCCTGAAC
AtSOC1-F	GTGTCAAATGTATTCGAGCAAG
AtSOC1-R	GAAGAACAAGGTAACCCAATGA
AtAP1-F	CTGTGATGCTGAAGTTGCTC
AtAP1-R	TGTATTGACGTCGGACTCAG
AtUBQ-F	AGGACAAAGAGGGTATCCCA
AtUBQ-R	CAGACGCAAGACCAAGTGAA

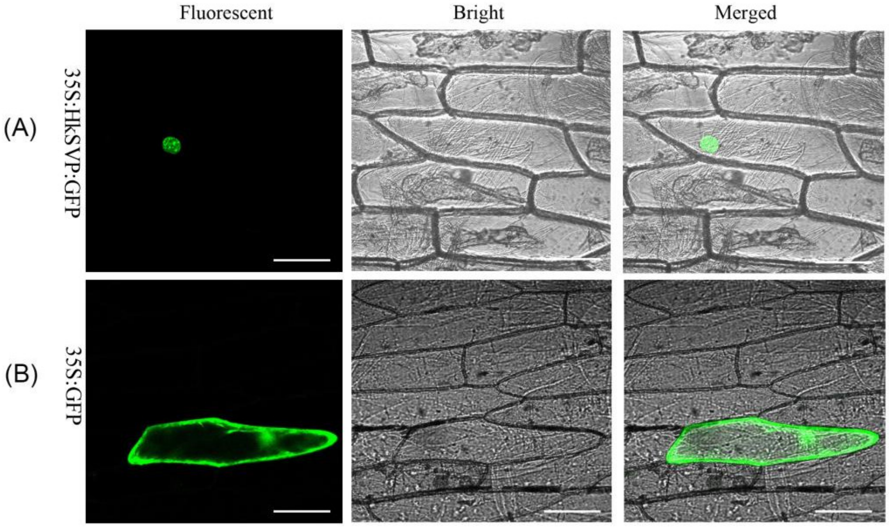

Figure S1. Subcellular localization of HkSVP in onion epidermal cell. A is HkSVP fused GFP at C terimal, B is GFP alone, which were both driven by the CaMV 35S promoter and introduced into onion epidermal cells by biolistic transformation. The transformed samples were maintained at 25°C for 24 h to observe fluorescent using the confocal microscope. Scale bars, 100 mm.

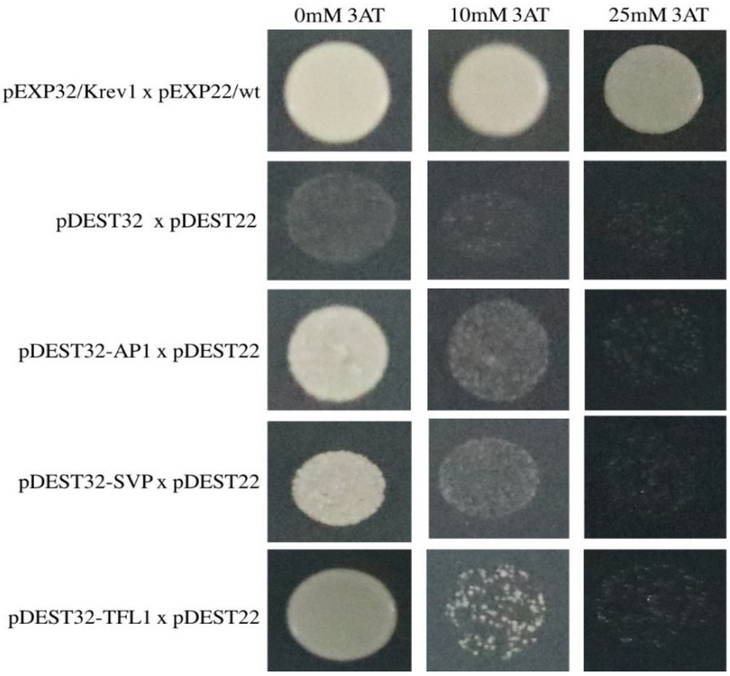

Figure S2. Detection of bait plasmid self-activation. 0 mM, 10 mM and 25 mM 3AT were added to the triple drop out (SC/-Leu/-Trp/-his) medium respectively to observe the growth of bait plasmid and control. All co-transformed combinations could grow at a lower 3AT concentration. When the 3AT concentration was 25 mM, the growth of bait plasmid was completely inhibited, Therefore, 25 mM 3AT was used as the screening concentration to inhibit the growth of false-positive yeast transformants.
